# Pathologic Femur Fracture in an Immunocompetent Healthy Young Adult due to Acute Osteomyelitis

**DOI:** 10.1155/2023/6279174

**Published:** 2023-12-04

**Authors:** Evan R. J. Goyette, Natalia Georgantzoglou, Darcy A. Kerr, Yvonne Cheung, Eric R. Henderson, Konstantinos Linos

**Affiliations:** ^1^Department of Pathology and Laboratory Medicine, Dartmouth-Hitchcock Medical Center, Lebanon, NH, USA; ^2^Geisel School of Medicine at Dartmouth, Hanover, NH, USA; ^3^Department of Radiology, Dartmouth-Hitchcock Medical Center, Lebanon, NH, USA; ^4^Department of Orthopedic Surgery, Dartmouth-Hitchcock Medical Center, Lebanon, NH, USA; ^5^Department of Pathology, Memorial Sloan Kettering Cancer Center, New York, NY, USA

## Abstract

An immunocompetent 33-year-old woman presented with a pathologic femur fracture after one month of progressively worsening right thigh pain. Open biopsy demonstrated acute suppurative osteomyelitis despite the lack of clinical risk factors. The polymicrobial infection was successfully treated with three operative procedures and culture-specific antibiotic agents. Acute osteomyelitis, while an uncommon cause of pathologic fracture, must always be on the differential diagnosis, even when no obvious predisposing factors are present. When investigating for an infectious etiology in cases such as our own, considering immunodeficiency syndromes alongside the more typical causes of osteomyelitis is encouraged.

## 1. Introduction

Acute osteomyelitis, an infection caused by pyogenic pathogens that leads to progressive bone destruction, is an uncommon disease of the immunocompetent adult without medical comorbidities. When it does occur, it tends to arise following direct traumatic inoculation or via contiguous spread from an adjacent soft tissue infection, as the risk for hematogenous bacterial seeding is greatly reduced upon epiphyseal closure at puberty [1–3].

This infection typically presents with localized discomfort, swelling, and erythema, whereas systemic signs occur in some cases. Laboratory studies typically reveal leukocytosis with elevated erythrocyte sedimentation rate (ESR) and C-reactive protein (CRP). Radiographs can exhibit soft tissue swelling with osteolysis and varying periosteal reactions, although radiographic changes in the bone are usually a delayed finding. Importantly, radiography can sometimes demonstrate aggressive permeative bone changes that overlap with high-grade malignancy. The gold standard for diagnosis is histopathologic evaluation and culture of bone biopsy, which demonstrates neutrophilic infiltration of marrow spaces and occasionally congested, thrombosed vessels with microorganisms. This workflow also provides material for complete microbiologic work-up, namely, identification of the causative organisms and antibiotic sensitivity testing. Prompt and aggressive treatment, generally involving surgical debridement and irrigation of devitalized tissue in conjunction with targeted antibiotic therapy, is usually curative in those without underlying conditions [1–3].

Delays in the detection or treatment of acute osteomyelitis can result in a host of complications, including pathologic fracture, whose most common cause in adults is occult malignancy [1, 4, 5]. To further complicate matters, pathologic fracture can rarely be the presenting symptom of acute osteomyelitis, a diagnosis that is often low on the differential when no risk factors or clinical evidence of infection are present [4, 5]. Herein, we report the first case of pathologic femur fracture caused by acute osteomyelitis in an immunocompetent 33-year-old woman with no apparent risk factors.

## 2. Case Report

**Figure 1:**
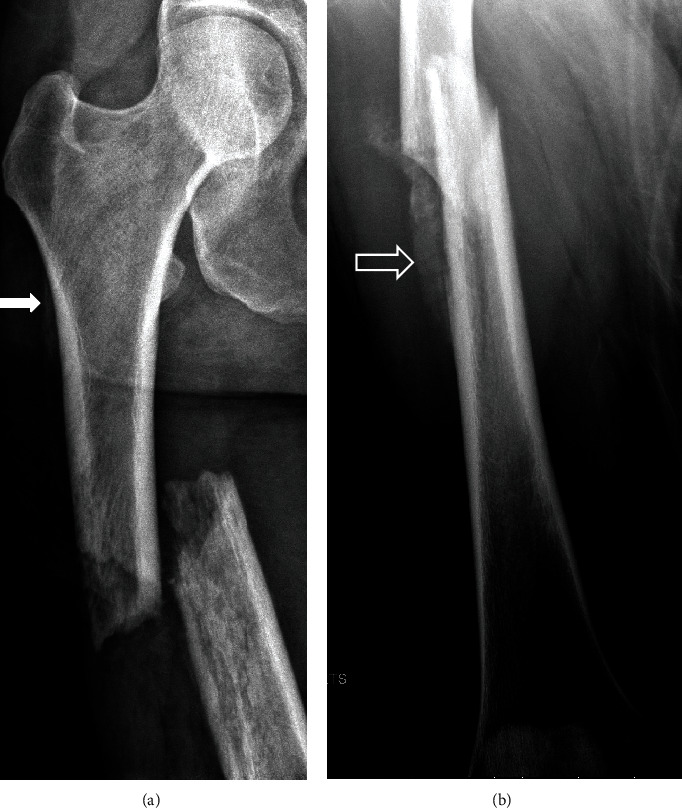
33-year-old woman with one month of vague thigh pain presented with pathologic fracture. (a) AP radiograph of the right femur shows a displaced pathologic fracture of the proximal femoral shaft. Smooth periosteal reaction (arrow) surrounds the proximal femoral shaft. Permeative bone destruction is seen at the fracture bone ends. (b) Oblique radiographic view shows additional organized, thick, and dense periosteal reaction or involucrum around the infected cortex (open arrow).

**Figure 2:**
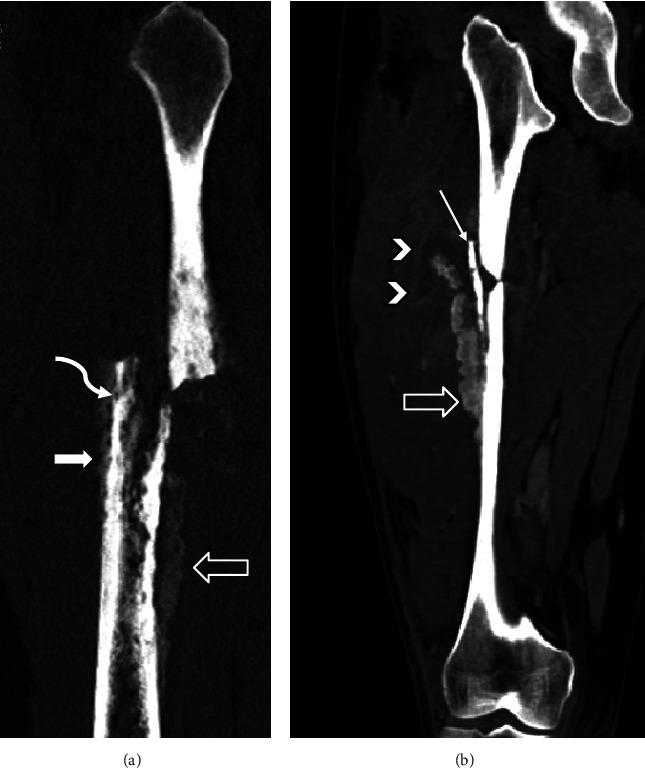
CT scan of right femur in the same patient 3 days after fracture. (a) Sagittal reformat of noncontrast CT image shows both organized (open arrow) and disorganized periosteal reactions (white arrow). The permeative bone destruction is best depicted on CT (curved arrow). (b) Coronal reformat of contrast CT image shows a low-density collection/lesion with a thin enhancing rim (arrowhead) around the fracture. The organized thick periosteal bone formation (open arrow) surrounds a very dense spicule that resembles necrotic bone or sequestrum (long thin arrow).

A 33-year-old woman with a history of alopecia areata treated with intralesional triamcinolone and multiple episodes of Herpes zoster exacerbation presented with one month of vague bilateral thigh pain, worse on the right side, for which she was originally diagnosed with piriformis syndrome and treated with meloxicam and physical therapy. While her left thigh pain dissipated, her right-sided pain progressed to the extent that weight-bearing was barely tolerable. She presented after experiencing an audible crack, immediate pain in her right leg, and collapse during ambulation. Of note, she endorsed an unintentional five-pound weight loss in the last month which she attributed to a loss of appetite secondary to pain. She did not experience numbness, tingling, or weakness in her affected leg, nor did she report fevers, night sweats, or new-onset fatigue. Remaining review of systems was negative, including no recent dental procedures. On exam, her right lower extremity was shortened, internally rotated, and tender, with erythema and swelling of the right midthigh, and intact neurovascular systems distal to the deformity. Her oropharynx was clear and without lesions. She had a leukocytosis of 11.5 with an absolute neutrophilia of 9.03 and a mild absolute monocytosis of 1.0, alkaline phosphatase of 151, ESR of 54, and CRP of 158.4. Testing for HIV was negative. She was empirically treated with vancomycin and ceftriaxone for presumed sepsis. Radiographs demonstrated a pathologic fracture of the right femoral shaft, at the site of permeative bone destruction (Figures 1(a) and 2(a)). There was both organized and nonorganized periosteal new bone formation (Figures 1(b), 2(a), and 2(b)). These were better characterized on noncontrast computed tomography (CT) images performed 3 days after the radiographs. The contrast CT images showed a hypodense collection or lesion in the vastus muscles surrounding the fracture. Imaging exams suggested a process with aggressive bone destruction surrounded by indolent periosteal reaction. Subsequent CT-guided biopsy of the bones around the pathologic fracture revealed acute osteomyelitis on microscopic examination. The differential diagnosis comprised neoplasia, including primary bone lesions, hematologic malignancy, and metastatic disease, as well as subacute bone infection.

**Figure 3:**
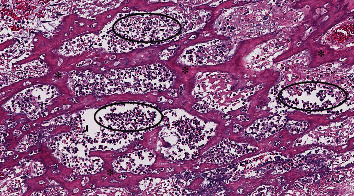
Right femoral bone biopsy demonstrating acute suppurative osteomyelitis, with abundant neutrophils (circles) present alongside a network of reactive woven bone (^∗^) (hematoxylin and eosin; 10x magnification).

**Figure 4:**
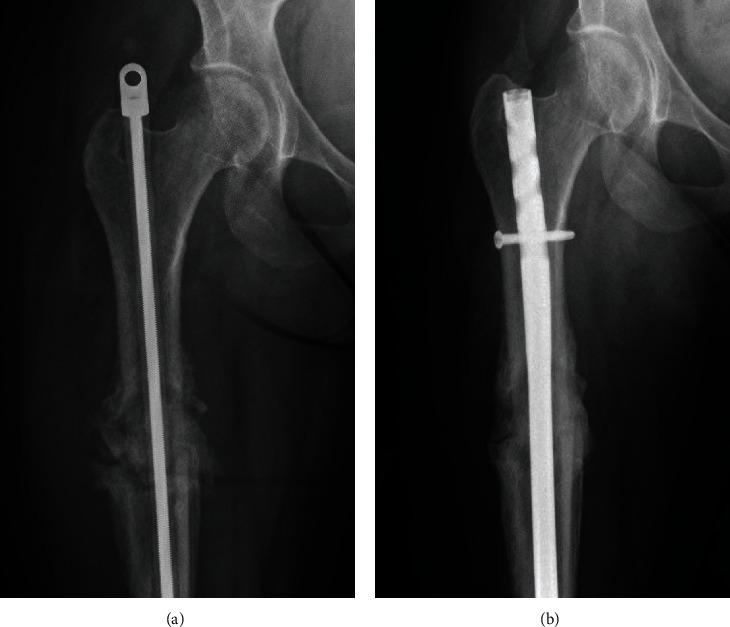
(a) Radiograph of right femur status post intramedullary fixation with an antibiotic-coated nail, approximately one month after fracture. There is significant increased bridging bone at the site of the prior pathologic fracture of the proximal right femoral shaft, consistent with fracture healing. (b) Radiograph of right femur status post intramedullary nail exchange and definitive fixation, approximately six months after fracture. Bony callus formation is evident in the region of the fracture with unchanged alignment.

Urine cultures grew methicillin-sensitive *Staphylococcus aureus* (MSSA) while blood cultures demonstrated *Streptococcus milleri*. Given the unexpected finding of acute osteomyelitis and the continued suspicion of underlying neoplasia, open biopsy was recommended; operative examination demonstrated a malodorous fracture hematoma with frank purulence and necrosis of bone and surrounding soft tissue, without gross evidence of tumor. Acute suppurative osteomyelitis was again evident microscopically (Figure 3). Cultures taken from the fracture site grew *Streptococcus milleri*, concordant with the organisms found in blood, along with MSSA, *Aggregatibacter aphrophilus*, and *Micrococcus* spp. Vancomycin was subsequently withdrawn from her antibiotic regimen. The patient then returned to the operating room for incision and debridement with intramedullary placement of a tobramycin/vancomycin-coated nail. Three days later, during operative nail exchange, the fracture site was no longer purulent. Her postoperative course was unremarkable, and she was discharged with instructions to maintain nonweight-bearing status on her right lower extremity for six weeks. She experienced no adverse events during her initial outpatient antibiotic course consisting of six weeks of intravenous ceftriaxone. Interval radiography showed evidence of callus formation, so she proceeded to undergo an uncomplicated exchange of her antibiotic-coated rod for a definitive implant (Figure 4(a)). While there was no intraoperative evidence of infection, the reamings from this procedure unexpectedly grew MSSA in broth culture, prompting the addition of three months oral doxycycline. After six months of follow-up, the patient was discharged from orthopedics, with radiographic demonstration of fracture healing and resumption of normal, unrestricted activity (Figure 4(b)). Immunologic and rheumatologic work-ups were pursued in the outpatient setting as well, given the suspicion for underlying causative immunodeficiency or autoimmune disorder. To date, thirty-nine months postfracture, this investigation has not identified a particular etiology that would predispose her to polymicrobial acute osteomyelitis. Specifically, her immunoglobulin levels were within the reference intervals, as was her CRP. No antinuclear antibodies were detected. During this follow-up, the patient disclosed that her most recent dental examination had occurred four months prior to her presentation.

## 3. Discussion

Risk factors for the development of acute osteomyelitis can be conceptualized by the different pathogenic mechanisms of the disease. Osteomyelitis that occurs via direct inoculation tends to result from open fractures, bites, and invasive medical procedures. Infection via contiguous extension from adjacent soft tissue abscess is usually the consequence of inadequate management of the precipitating pathogen. Vascular compromise due to diabetes mellitus, peripheral vascular disease, and hemoglobinopathy exacerbates the risk of contiguous spread. These comorbidities also increase the likelihood of hematogenous dissemination to distant sites, as disruption of microvascular integrity allows the causative microbe to enter the bloodstream. Vascular access and subsequent bacteremia can also be caused by indwelling catheters or distant foci of visceral infection, including respiratory, gastrointestinal, or genitourinary. Additionally, nonpenetrating trauma can act as a nidus for bacterial colonization, as the resultant soft tissue hematoma is a rich medium for microbial proliferation [2, 6].

The existing English-language medical literature demonstrates few cases of acute osteomyelitis presenting with pathologic femur fracture, with all cases having identifiable risk factors [4, 5, 7–9]. Despite extensive review of this young woman's medical record, no risk factors could be identified. While both urine and fracture cultures grew out MSSA, the absence of MSSA bacteremia argues against urinary tract infection as the source for hematogenous dissemination. The occurrence of an oral health procedure in the months preceding this infection raises the possibility of a dental source of infection. However, persistence of bacteremia over a period of months would be unusual, as in the immunocompetent host, this is typically cleared within 20 minutes after completion of a dental procedure [10]. Furthermore, the polymicrobial nature of her condition is unusual for hematogenous osteomyelitis, which is classically monobacterial [2]. Certainly, *S. milleri*, part of the endogenous flora of the oropharyngeal, gastrointestinal, and genitourinary tracts, could have seeded the femur initially following transient bacteremia or other clinically silent disease process. However, the congregation of the remaining identified species, which are not all localized to a single body cavity, is curious.

One plausible explanation would be an underlying immunodeficiency. In their recent summary of the skeletal and joint manifestations of primary immunodeficiencies, Gharib and Gupta found osteomyelitis to be the most common diagnosis [11]. Meanwhile, Xu et al. recently described a case of *Mycobacterium avium* scalp osteomyelitis caused by anti-interferon-*γ*-associated immunodeficiency [12]. Clearly, in cases such as this where obvious risk factors and etiologies are unclear, consideration of immunodeficiencies, both primary and secondary, with full immunologic work-up is warranted.

Acute osteomyelitis should always be considered in the differential diagnosis of pathologic fractures. Consideration of the different pathogenic mechanisms of osteomyelitis can be helpful when the cause of the disease is uncertain. In such cases, thinking broadly to include immunologic conditions alongside the more classic explanations of osteomyelitis is recommended.

## Data Availability

The data that support the findings of this study are available from the corresponding author upon reasonable request.

## References

[1] Schmitt S. K. (2017). Osteomyelitis. *Infectious Disease Clinics of North America*.

[2] Lew D. P., Waldvogel F. A. (2004). Osteomyelitis. *The Lancet*.

[3] Hatzenbuehler J., Pulling T. J. (2011). Diagnosis and management of osteomyelitis. *American Family Physician*.

[4] Dick H. M., Rosenberg A. E. (1995). Case 26-1995 — A previously well, 29-year-old woman with a pathologic fracture of the femur. *New England Journal of Medicine*.

[5] Gelfand M. S., Cleveland K. O., Goswami R., Heck R. K. (2006). Pathological fracture in acute osteomyelitis of long bones secondary to community-acquired methicillin-resistant *Staphylococcus aureus*: two cases and review of the literature. *The American Journal of the Medical Sciences*.

[6] Wald E. R. (1985). Risk factors for osteomyelitis. *The American Journal of Medicine*.

[7] Krebs N. M., Krebs R. C., Yaish A. M. (2017). Femoral osteomyelitis presenting as a pathologic fracture in a 53 year old male: a rare case report. *Journal of Orthopaedic Case Reports*.

[8] Thein R., Tenenbaum S., Chechick O., Leshem E., Chechik A., Liberman B. (2013). Delay in diagnosis of femoral hematogenous osteomyelitis in adults: an elusive disease with poor outcome. *The Israel Medical Association Journal*.

[9] Janssen E. H. C. C., de Bree L. C. J., Kant K. M., van Wijngaarden P. (2017). Spontaneous fracture of the femur due to osteomyelitis caused by the *Streptococcus anginosus* group. *The Netherlands Journal of Medicine*.

[10] Martins C. C., Lockhart P. B., Firmino R. T. (2023). Bacteremia following different oral procedures: systematic review and meta-analysis. *Oral Diseases*.

[11] Gharib A., Gupta S. (2016). Skeletal and joint manifestations of primary immunodeficiency diseases. *SOJ Immunology*.

[12] Xu X., Lao X., Zhang C. (2019). Chronic *Mycobacterium avium* skin and soft tissue infection complicated with scalp osteomyelitis possibly secondary to anti-interferon-*γ* autoantibody formation. *BMC Infectious Diseases*.

